# Automatic versus Voluntary Motor Imitation: Effect of Visual Context and Stimulus Velocity

**DOI:** 10.1371/journal.pone.0013506

**Published:** 2010-10-20

**Authors:** Ambra Bisio, Natale Stucchi, Marco Jacono, Luciano Fadiga, Thierry Pozzo

**Affiliations:** 1 Department of Robotics, Brain and Cognitive Sciences, Italian Institute of Technology, Genova, Italy; 2 Dipartimento di Informatica, Sistemistica e Telematica, Università degli Studi di Genova, Genova, Italy; 3 Dipartimento di Psicologia, Università degli Studi di Milano-Bicocca, Milano, Italy; 4 Dipartimento di Scienze Biomediche e Terapie Avanzate-Sezione di Fisiologia Umana, Università degli Studi di Ferrara, Ferrara, Italy; 5 Institut National de la Santé et de la Recherche Médicale-U887, Motricité-Plasticité, Campus Universitaire, Dijon, France; The University of Western Ontario, Canada

## Abstract

Automatic imitation is the tendency to reproduce observed actions involutarily. Though this topic has been widely treated, at present little is known about the automatic imitation of the kinematic features of an observed movement. The present study was designed to understand if the kinematics of a previously seen stimulus primes the executed action, and if this effect is sensitive to the kinds of stimuli presented. We proposed a simple imitation paradigm in which a dot or a human demonstrator moved in front of the participant who was instructed either to reach the final position of the stimulus or to imitate its motion with his or her right arm. Participants' movements were automatically contaminated by stimulus velocity when it moved according to biological laws, suggesting that automatic imitation was kinematic dependent. Despite that the performance, in term of reproduced velocity, improved in a context of voluntary imitation, subjects did not replicate the observed motions exactly. These effects were not affected by the kind of stimuli used, i.e., motor responses were influenced in the same manner after dot or human observation. These findings support the existence of low-level sensory-motor matching mechanisms that work on movement planning and represent the basis for higher levels of social interaction.

## Introduction

Motor imitation, that is, the possibility of interacting physically with others by sharing a behavioural state, represents a powerful biological resource for cognitive development [1] and social interaction [2]. In these cases, imitation occurs via automatic processes, whereas examples of voluntary imitation are experienced daily while learning new tasks (learning by imitation [3]). Despite the literature widely treating this topic, the difference between voluntary and automatic imitation remains unclear, and the role of awareness in transforming the visual input provided by a model into a motor command produced by the observer has not been extensively investigated.

This topic is controversial and became the matter of different theories of imitation that pose either high-level (Goal-Directed Imitation theory, GOADI [4]) or lower-level order mechanisms (Direct Matching Hypothesis [5] and Ideomotor Framework of Imitation [6]) at the base of the sensory-motor transformation. While the Direct Matching Hypothesis and the Ideomotor Framework of Imitation agree on the fact that when individuals see external actions and their consequences, they activate representations of their own actions that would produce those same consequences, the GOADI theory supports the idea that imitation is guided by cognitively specified goals.

However, when a goal is difficult to extract from the visual scene, one can speculate that kinematics may be helpful to infer it. Since a demonstration by a model is mainly a spatiotemporal time-varying event, one can thus predict that imitation might be responsive to the kinematics characterizing the observed motion. For instance, even a poor visual stimulus, like a point-light display of a walker, is recognizable as a human body as soon as it starts to move [7]. The sensibility of visual perception to action kinematics was also demonstrated by Pozzo et al. [8] during an inference task in which people were required to infer the final position of a simple dot moving with a biological or non-biological trajectory. Similarly, Noy et al. [9] and Bove et al. [10] proposed that observers use the kinematics of stimuli rather than their pictorial description to map perceived movement onto executed movements. At the same time, other researchers have focused on the importance of the visual context for action recognition [11], [12] drawing attention to the presence of a meaningful visual scene (e.g., a picture, a video or an individual, interacting intentionally with an object or another person) vs. a meaningless one as significant cause of difference in an observer's motor performance.

In this context, the present study examines the motor effects of intentional and automatic imitation. More specifically, we want to verify if kinematics (here, this term refers to an action's spatiotemporal/time-varying characteristics) can tune the imitation process. In particular, the questions we want to answer are the following: 1) is the actual movement production influenced by the vision of a prior biological moving stimulus, leading to a spontaneous contamination? 2) If this is the case, what is the difference between implicit (automatic) and explicit (voluntary) imitation, and how sensitive are they to visual stimuli (abstract vs. human)? 3) Does the biological kinematic tune automatic speed contamination? 4) Are observers able to reproduce exactly the velocity of a visual stimulus when they are explicitly asked to imitate it? To these aims, we compared voluntary imitation performances to the motor features of pointing tasks both executed after the display of moving visual stimuli, differing in velocity (slow, medium, and fast speeds), in kinematic (biological vs. non-biological) and in shape (dot vs. human demonstrator).

## Materials and Methods

This study was divided into three parts hereafter called Preliminary, Movement Observation and Kinematic experiments. A total of nineteen healthy young adults participated in the experiments. All participants were right-handed according to an informal interview, and had normal or corrected-to-normal vision. Written informed consent was obtained from each participant in the study, which was approved by the local ethical committee ASL-3 (“Azienda Sanitaria Locale”, local health unit), Genoa, and was in agreement with the Helsinki Declaration of 1975, as revised in 1983.

### Materials and Procedure

#### Preliminary experiment

The preliminary experiment was aimed at measuring participants' natural pointing movements. The kinematic data served as a baseline to be compared with arm kinematics after motion observation. Fourteen healthy participants (8 men and 6 women, age 25±2) took part in this experiment.

Apparatus. The experiment was performed in a darkened room. Participants sat on a chair, in front of a large rear projection screen (190×140 cm) at a viewing distance of about 90 cm. A video-projector, with a refresh rate of 75 Hz, placed behind the screen and connected to a PC, back-projected the visual stimuli onto the display screen. A VICON Motion Capture System with seven cameras was used to record movements at a sampling frequency of 100 Hz. One passive infrared reflective marker was applied onto a fingertip of each participant's right hand.

Stimuli, tasks, and procedure. Two vertically aligned light blue dots (2 cm in diameter), placed at a distance of 72 cm from each other were displayed for 300 ms on the screen. One of the two dots served to show the starting position of the participant's arm and the other one was the target for the movement. The participants' shoulder level was roughly at the middle of the dots. Participants had to perform upward and downward single arm movements with their right arm extended, at a spontaneous, natural velocity, and without making any final corrections (i.e. one-shot movements). Before each trial, participants were verbally informed of the starting position of the subsequent movement, and they were instructed to point there with their right finger. When both dots disappeared, the subjects moved their arm up to the memorized position of the target dot. Movement accuracy was not emphasized at all. For each direction the pointing arm movement was replicated 10 times in a random order. The beginning of the experiment was preceded by a training phase of 5 movements for each direction.

#### Movement Observation experiment

A moving stimulus was used as a template to test the effect of motion perception on subsequent arm movement execution. Participants were the same of the Preliminary experiment.

Stimuli, tasks, and procedure. Participants accomplished two types of tasks: implicit (I) and explicit (E). They differed only in the instructions given to the participants: during implicit task they were free to produce movements at a velocity of their choice; whereas during explicit task subjects were requested to imitate the stimulus motion velocity. Each task was divided into two conditions in which either a dot (D) or a human (H) was used as the stimulus.

**Figure 1:**
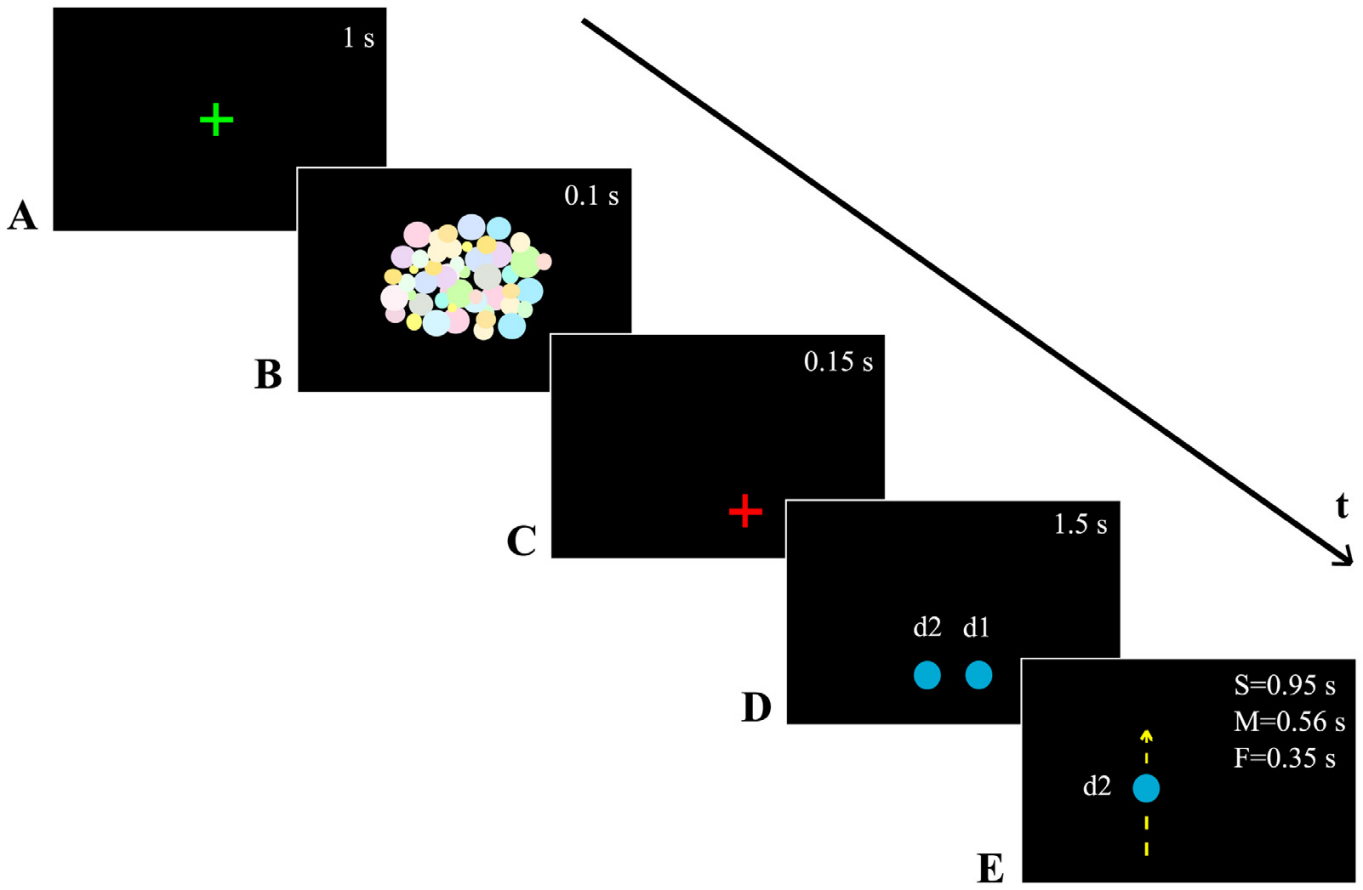
Sequence of visual stimuli. *A*. A green cross was the alert signal that a new trial was going to start. *B*. 400 random disks different in size, colour, and position, appeared. *C*. a red cross was displayed at the starting position of the movement for 150 ms. *D*. The red cross was substituted by d1 and d2 appeared on the left of d1 at the same time. *E*. d1 disappeared when d2 started to move upwards (as in this figure) or downwards. The white numbers in each box indicate the duration of the associated stimuli. In *D* the duration varies with respect to the experimental condition. The dimensions of the stimuli in this figure do not respect the real dimensions.

Dot observation. The Apparatus was the same as in the Preliminary experiment. The stimuli sequence is shown in Figure 1. Participants observed single upward and downward motions of a blue dot 2 cm in diameter. The motion's presentation was included in a sequence of visual stimuli generated using MatLab Psychtoolbox 3 [13]. The appearance of a green cross at the centre of the screen (Figure 1A) cued the participants for the beginning of a new trial, which always started with a 100 ms mask (Figure 1B). The mask was composed of two-dimensional randomly distributed small discs, randomly variable in luminance and colours (with diameters between 2 and 20 cm). The mask covered a circular area of about 100 cm in diameter. After presentation of the mask, a red cross was displayed at the starting position of the movement (Figure 1C). After 150 ms, the red cross was replaced by a blue dot (d1) with a second dot (d2) on its left (Figure 1D). Participants had to point to d1 to avoid having d2 covered by their right arm. When d2 started its motion d1 disappeared (Figure 1E). Participants did not know if the dot's motion was computer- or human- generated. The motion of d2 (72 cm covered) respected the kinematics of a vertical arm-pointing movement (biological motion). In actuality, upwards- and downwards- pointing arm movements are not identical. Their velocity profile is, of course, roughly the same: zero velocity at start, acceleration phase, peak of velocity, deceleration phase, and stop. In contrast to analogous horizontal pointing movements [14], the velocity profiles of vertical movements are asymmetric: though they share the same duration, upward displacement has a shorter acceleration phase than that of downward displacement [15]. Dot motions differed in velocity: slow (S), medium (M), and fast (F). Mean dot velocity values are reported in Table 1 (columns DI and DE). Stimulus directions and velocities were randomized. Participants were asked to point at the red cross/d1, then to watch the movement of d2 and to reach the level at which d2 vanished, in both tasks. Pointing movements were to be one shot (i.e. without final adjustments) and parallel to the trajectory of d2. Thus, the executed movements were congruent with the observed ones in terms of direction. Each dot motion velocity was repeated 8 times.

**Table 1:** Stimuli and participants' movement mean velocities [m/s].

	UP				DOWN			
**N**	0.81±0.16				0.78±0.2			
	**DI**	**I**	**DE**	**E**	**DI**	**I**	**DE**	**E**
**S**	0.74	0.75±0.2**	0.74	0.56±0.1 **	0.74	0.72±0.2**	0.74	0.51±0.1**
**M**	1.25	0.86±0.2**	1.25	0.82±0.2	1.25	0.82±0.3**	1.25	0.83±0.2*
**F**	2	0.93±0.3**	2	1.17±0.2 **	2	0.9±0.3 **	2	1.16±0.2**
	**HI**	**I**	**HE**	**E**	**HI**	**I**	**HE**	**E**
**S**	0.79±0.2	0.74±0.*	0.84±0.3	0.54±0.1 **	0.73±0.2	0.76±0.3	0.75±0.2	0.48±0.2**
**M**	1.43±0.3	0.85±0.2	1.42±0.3	0.9±0.24 *	1.45±0.3	0.84±0.3	1.43±0.3	0.85±0.3
**F**	2.09±0.4	0.96±0.3 **	2.03±0.5	1.26±0.3 **	2.12±0.4	0.92±0.3**	2.19±0.4	1.26±0.3 **

The second line indicates the participants' natural velocities (N) for upward and downward movements. The columns DI, DE, HI and HE report stimuli velocities for each experimental condition (Slow, Medium and Fast). While dot (D) velocity was always the same, the demonstrator's velocity (H) changed with the conditions. The white columns give the participants' mean velocity values in implicit (I) and explicit (E) tasks. The star (*) indicates a statistically significant difference between the side value and the natural velocity (N), in the same movement direction (* p<0.05, ** p<0.01).

Human observation. The person that acted as the stimulus (hereafter called the demonstrator) was a woman and was the same in all the experiments. She was previously trained to make a one-shot, straight, vertical pointing movement at three different velocities as close as possible to the dot velocities (slow, medium, and fast). The demonstrator's mean velocities for each experimental condition are reported in Table 1 (columns HI and HE). The demonstrator and participant faced each other, and the participant was to mirror the demonstrator's arm movements. In both the implicit and the explicit tasks, participants were instructed to point at the demonstrator's fingertip, then to look at it until its movement stopped and to arrive at their own fingertip's final position with a one-shot movement. A random order of trials was prepared at the beginning of the experiment. The demonstrator followed this order in executing her movements but participants were completely unaware of this. Both the participants' and the demonstrator's movements were recorded with the VICON motion capture system.

Experimental design. Four within-subjects factors were considered as sources of variability: Task (implicit, explicit), Stimulus (dot, human), Direction (up, down), and Velocity (slow, medium, fast), resulting in a total of 192 trials (8 replications per condition). After a block of 16 trials, participants took a pause for at least one minute. The experimenter reminded them of the instructions after each pause. The experiment was preceded by a training phase (6 movements: 3 velocities x 2 directions) for each task and stimulus. The implicit task always preceded the explicit one in order to keep the subjects unaware of the latter task during implicit condition. Performing the explicit before the implicit task could lead the subjects to imitate explicitly, regardless of the experimental requirements (to imitate or only to reach the target).

#### Kinematic experiment

A dot moving according to a non-biological kinematic was used to figure out the role of the kinematic features in speed contagion. Five participants (3 men and 2 women, age 26.6±1.14) took part in the experiment. No one of them performed Preliminary and Movement Observation experiments.

Stimuli, tasks, and procedure. Because our main interest was on the automaticity of movement planning, participants accomplished only the implicit (I) task: thus, they were free to produce movements at a velocity of their choice. Moreover, because of the impossibility to force a human demonstrator to violate the biological law of motion, the dot was the only stimulus used. The apparatus and the stimuli sequence were the same as in the Movements Observation experiment (for the stimuli see Figure 1 and Table 1). The dot moved according to a uniformly accelerated motion, thus violating the biological laws. This kinematic was chosen against other possibilities (e.g. constant velocity) because, as in biological condition, the motion started by an acceleration. Therefore, the only difference between Kinematic and Movement Observation experiments was dot's velocity profile.

### Data treatment

#### Data processing

Data were low-pass filtered at 5 Hz using a 2^nd^ order Butterworth filter. To define the onset and offset of the movement, we chose a threshold corresponding to 5% of the maximum value of the movement velocity profile. The same processing methods were applied to analyze the pointing movements of both the participant and the demonstrator.

#### Data analysis

Preliminary experiment. A paired t-test was used to compare mean movement velocity (V) values in upward and downward pointing movements.

Movement Observation experiment. For all trials, V was considered as a main outcome variable. Outlier values (more than twice the standard deviation) were removed from the analysis. In order to determine the role of the observed motions in movement execution, we compared V (Slow, Medium and Fast) with the baseline values (Preliminary experiment, natural velocity) by mean of a paired t-test, with Dunnett correction for multiple comparisons. After that, stimuli (dot and human) V were statistically compared. As reported below, because of the difference between their kinematic features, we analyzed the participants' responses to dot and human stimuli separately, applying two mixed-model analyses of variance on V, one for each stimulus. These allowed for detecting any systematic effects of Task, Direction, and Velocity. This statistical analysis method was chosen for its flexibility to designs that are not perfectly balanced, as in our case. Moreover, it allows for taking into account the intrinsic (and uncontrolled) variability among the participants, which was considered everywhere as a random factor. This information will not be repeated hereafter in the text. Significant interactions between factors were examined with Post-Hoc Newman-Keuls comparisons. A linear regression model illustrated the relationship between stimuli and the participants' V values. The parameters of the linear fits describing the stimuli and the participants' V relationship were mainly used to compare the effects of the two stimuli on the participants' responses: the slopes and the intercepts of the regression lines obtained for each participant were evaluated statistically using three factors (Stimuli, Task and Direction) mixed model analysis.

Kinematic experiment. A one-way ANOVA was applied to test the effect of the stimulus velocity (factor Velocity). A linear regression model illustrated the relationship between the V values of stimuli and participants. In order to test the effect of the biological and the non-biological kinematics, we compared the slopes here obtained with those calculated in the Movement Observation experiment (factor Kinematic) through a one-way ANOVA.

## Results

### Preliminary experiment

**Figure 2:**
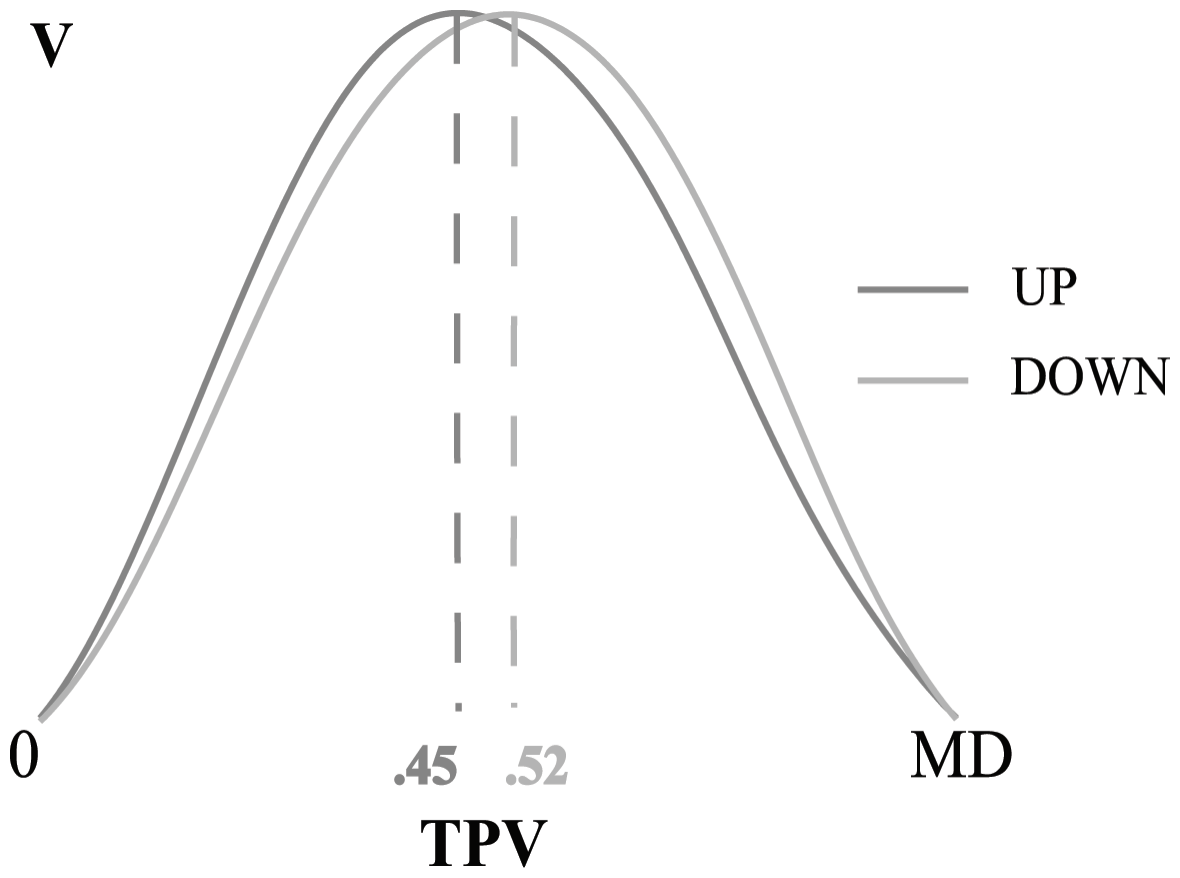
Preliminary experiment, velocity profile. Upward (dark grey) and downward (light grey) movement velocity profile of a typical subject, normalized on amplitude and duration (MD). On the bottom the Time to Peak Velocity values (TPV) of these movements are reported.

Modulations of velocity along the trajectory were in agreement with those already described by Papaxanthis et al. [15]: the time to peak velocity values (TPV) were 0.43±0.07 and 0.5±0.06 (mean ± standard deviation) for upward and downward movements, respectively. Figure 2A shows an example of an upward and downward velocity profiles for a typical subject. The mean TPV values of participants in the two directions were statically different (*F*(1,15) = 37.89, *p*<0.01), whereas the mean velocities (V, up: 0.81±0.16 m/s, down: 0.78±0.2 m/s) were not.

### Movement observation experiment

This experiment was aimed at testing to which degree the velocity of an executed arm movement mimicked the velocity of an observed motion. Pointing movements were spontaneous or constrained to imitate previously observed motions (Task factor: implicit and explicit, respectively). We tested if participant's movements were influenced by the stimuli mean velocity. In order to control against possible contamination, the participants' mean velocity values (V) were examined. In order to determine how much participants modified their natural movement velocity (see Preliminary experiment) after observing a moving stimulus, V, at each level of the Velocity factor, was compared with each participant's baseline velocity using a paired t-test. Table 1 reports the participants' V values for each experimental condition (white columns). The star (*) on the right side indicates a significant difference from the natural pointing velocity. Significant differences were present both in explicit and in implicit tasks, demonstrating that the participants' velocities were influenced by previously observed motions, even when no constraints in velocity were given by the experimenter.

**Figure 3:**
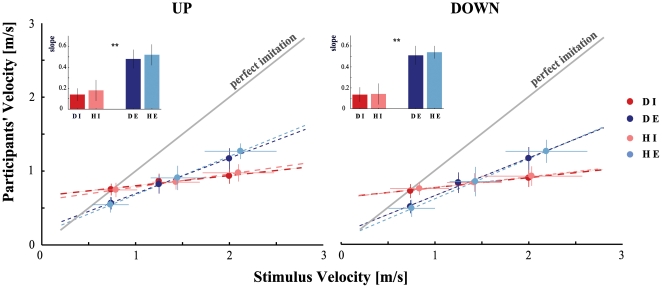
Linear relationship between participant (*y*-axis) and stimuli velocities (*x*-axis). Left and right panels refer to upward and downward pointing movements, respectively. The colours code the Task and the Stimulus observed. The red scale refers to implicit task (I) and the blue scale refers to explicit task (E). Light colours represent responses to the human (H) and dark colours to the dot (D). The *y = x* grey line indicates the theoretical perfect imitation of the stimulus motion velocity: data above (below) this line correspond to an overestimation (underestimation) of the observed movement velocity. The vertical error bars represent standard deviations. It can be noted that the demonstrator velocity was actually inaccurate in reproducing the dot's velocities (see the horizontal error bars). The dashed lines represent the results of the linear regression model applied on the data for each experimental condition. The two insertions represent slope values (*y*-axis) and statistics. ** indicates a statistically significant effect (*p*<0.01) of the Task factor (I vs. E), regardless of the stimuli presented.

Figure 3 shows the mean velocity of the participants' movements after observation of a dot (dark colours) and of a human demonstrator (light colours), for both implicit and explicit tasks, and in both directions. Upon a visual inspection of the data, the modulation exerted by stimuli velocities on participants' movements appears evident: the slowest and fastest arm pointing movements were always preceded by slow and fast stimuli motions, respectively. A positive linear relationship between these two quantities emerged, and the similarity between the regression lines of the two stimuli appeared evident, in both tasks and directions. Explicit slope values were higher than implicit ones. Below, we provide a statistical description of these results. A paired t-test compared the mean velocities of the two stimuli. As anticipated in the Materials and Methods section, the result showed a significant difference (p<0.01). Therefore, statistical analysis on the participants' mean velocities was performed separately for each stimulus. Two general mixed model analyses with three fixed factors (Task, Direction, and Velocity) were applied to V. For both stimuli, the analyses revealed a significant interaction between the Task and Velocity factors (Table 2A). This interaction did not affect the systematic effect of the Velocity because the same trend was observed overall. In contrast, it could hide a possible difference between the two levels of the Task. As expected, the Velocity factor was statistically significant, as opposed to the Task factor (Table 2A). Nevertheless, a Newman-Keuls post-hoc comparison exhibited a significant difference between implicit and explicit tasks, for both Slow and Fast conditions (Table 2B). In Slow (Fast) condition implicit velocity values were always higher (lower) than explicit ones, where the actual velocities more closely approached those of the observed motion. These results pointed out the fundamental role played by the experimenter's instructions on participants' performances. On the contrary, the movements' velocities were not significantly affected by their directions. For both tasks (implicit, explicit) and for both stimuli (dot, human), the actual velocities were linearly related to the stimuli velocities. A linear regression model (obtained using the MatLab Curve Fitting Tool) described these relationships for each participant (R^2^>0.8 in all conditions). Lastly, participants came closer in explicit task to retracing the line representing a perfect imitation of stimuli velocities (Figure 3, grey line), than they did in implicit task, though still failing to reproduce them exactly. Slopes values are represented in the two upper insertions in Figure 3. A mixed model analysis on the slopes and the intercepts of the best fitting lines (three fixed factors: Stimulus, Task, and Direction) proved the difference between the tasks (slopes: *F*(1,13) = 238.56, *p*<0.01; intercepts: *F*(1,13) = 182.39, *p*<0.01), whereas the Direction factor did not significantly affect the subjects' performance. Likewise, the Stimulus factor was not statistically significant, suggesting that differences in the qualitative features of the visual stimulation did not evoke different motor responses. Moreover, a statistical comparison of the slopes values between a non-imitative behaviour (horizontal line, slope  = 0) and the implicit task gave further proof of the implicit contamination applied by the visual stimuli. A paired t-test was used to compare up and down slopes of the implicit task with the null value of the horizontal line. A significant difference (p<0.01) confirmed the priming role of movement perception in action execution.

**Table 2:** Statistical analyses on the participants' mean velocity (V) values in the Movement Observation experiment.

A	DOT		HUMAN	
**Task**	F(1,13) = 0.07	p>0.05	F(1,13) = 0.96	p>0.05
**Vel**	F(2,26) = 319.74	p<0.01**	F(2,26) = 75.94	p<0.01**
Task*Vel	F(2,26) = 129.41	p<0.01**	F(2,26) = 97.28	p<0.01**

B	UP	DOWN	UP	DOWN
**S**	<0.01	<0.01	<0.01	<0.01
**M**	>0.05	>0.05	>0.05	>0.05
**F**	<0.01	<0.01	<0.01	<0.01

*A* shows a part of a mixed model analysis with three fixed factors (Task, Direction, and Velocity). The effect of the Velocity and Task factors and their interactions are here reported. The Direction factor is omitted because it was not significant and does not add any information. *B* reports the *p*-values from a Newman-Keuls post-hoc comparison on V in order to show the difference between implicit and explicit task for the three velocities and for both directions.

### Kinematic experiment

This experiment was performed to evaluate the changes in participants' performances after the observation of different stimuli kinematics. We predicted that speed contagion should be more pronounced for biological than for non-biological stimuli motions. This result would explain speed contagion as a phenomenon mediated by automatic imitation process, and not exclusively by visuomotor priming. Since no differences in Movement Observation experiment appeared between the two directions, participants performed only upward movements.

**Figure 4:**
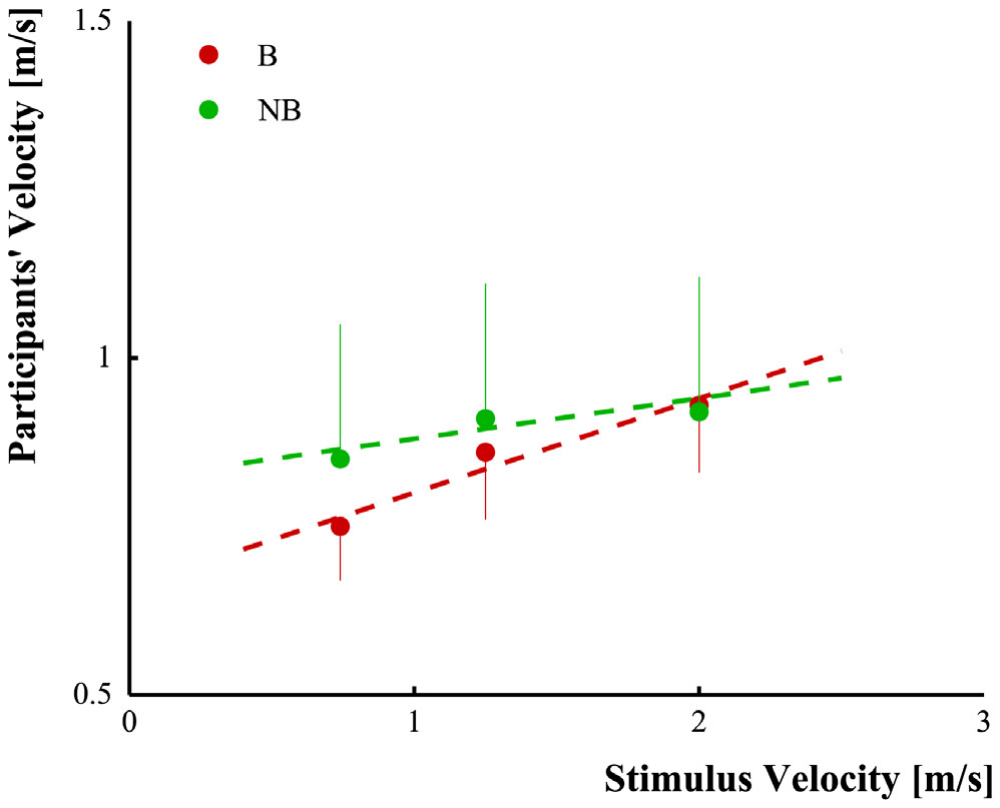
Differences in movement execution after the observation of biological (red circles) and non-biological (violating biological laws, green circles) motions: linear relationship between participant (*y*-axis) and stimuli velocities (*x*-axis) for upward movements in implicit task. The circles represent participants' movement velocities after observing the moving stimuli and the vertical error bars refer to the standard deviations values. The dashed lines are the results of the linear regression model applied on the data.

Figure 4 illustrates the results. The one-way ANOVA on participants mean velocity did not reveal any significant effect of the factor Velocity. Conversely, the one-way ANOVA on the slopes of the biological (slope = 0.14) and non-biological (slope = 0.06) linear fits revealed a significant effect of the factor Kinematic (*F*(1,17) = 8.09, *p* = 0.01). This indicated that participants' motor performances were affected by motion kinematics.

## Discussion

The present study analyzed the effect of moving visual stimuli, differing in shape (dot vs. human demonstrator) and in velocity (slow, medium, and fast speeds), on the kinematic features of a pointing task performed after the display of moving stimuli. First and foremost, the results indicate that the participants' pointing velocities varied as a consequence of the stimuli velocities, even when the participants were not explicitly asked to imitate the observed motion. Moreover, dot (an abstract and meaningless stimulus) and demonstrator' motions produced the same effect on subsequent participants' pointing movements. Even though imitative behaviour has been demonstrated in several situations, to the best of our knowledge, no study has described the effects of perception of a moving stimulus on action's kinematics. The following discussion will consider the issues of implicit and explicit imitation and the origin of the visual stimulus (human- or computer- generated) in the imitation process.

### Spontaneous tendency of the participants to imitate the velocity of the previously observed motion

This study addresses the topic of spontaneous imitation by considering the effect of visuomotor priming [16] on movement itself. Even when not required, participants automatically changed their natural velocity according to the stimulus velocity. When the latter was lower or higher than the participants' baseline velocity, subsequent pointing movements became systematically slower or faster than the natural ones. This result suggests that visual processing automatically induces related motor responses, namely, an implicit imitation of stimulus velocity. This finding strongly supports the previous assumption of automaticity in imitative behaviours [17]–[20], and supports the idea of direct matching between observed and executed motions. From literature, further proofs on the existence of automatic, mutual contagion between action observation and execution were provided by Jacobs et al. [21] and Watanabe [22]. While the first proved that the judgement of external motion varied when the observer's status changed (stationary or moving), the latter demonstrated that the observation of biological motions, differing in velocity, caused a contagion effect on the subjects' reaction times. In our case, the biological kinematics of both stimuli would be the key factor inducing this implicit speed modulation, as also suggested by the equal contribution of the dot and the human stimuli and by the difference between biological and non-biological kinematics (see following paragraph). In a recent study [23], Chong et al. tested the susceptibility of automatic imitation to selective attention and reported an increase in the reaction time required to initiate a congruent hand movement with a concurrently observed action. In contrast, the authors failed to show a motor effect, i.e. the transport time toward the object remained unaffected by the visual stimulus. Among the possible reasons, they suggested that the “interference between an observed and executed action manifests mostly during the early phase of action execution”. In contrast, our results demonstrate that a kinematic variable (the pointing velocity) was affected by prior observation of the moving stimulus. However, the discrepant results may be due to our moving stimuli, compared to the static stimuli (hand posture) used in [23] that might exclusively contribute to the selection of a specific hand posture but not to the reaching movement.

Moving the discussion toward a computational approach, the execution of a natural pointing movement requires a series of processes that seem to be based on the optimality principle [24], [25]. For the present pointing movement in the sagittal plane, the optimal control strategy based on energetic minimization [26] would produce the unconstrained pointing velocity recorded in the Preliminary experiment. In contrast, when observation precedes the pointing action, the cost function, i.e. the function to be minimized in the optimal control model, has to be weighted with external cues that may activate implicit mechanisms of action imitation. Interestingly, the linear relationship found between stimulus and participant movement velocities for both the implicit and explicit tasks suggests that automatic and voluntary imitations share a common mechanism where the *optimal module* (endogenously imposed) and *imitation constraints* (exogenously imposed) combine linearly. When the priority is to imitate the displayed movement (explicit condition), the weight of the imitation constraints is maximal. Instead, in implicit condition, the mechanism combining the optimal response with imitation constraints would produce a clockwise rotation of the explicit regression line towards the horizontal line (corresponding to the optimal pointing velocity) as observed here.

### Participants' performance did not change with human or abstract stimuli moving according to biological laws

Surprisingly, we did not find a better imitation of the human demonstrator as compared to the abstract stimulus. Regarding previous investigations using interference or SRC paradigms, this result was difficult to predict. For instance, it has been proposed that the biological relevance of the visual stimulus is the key feature in eliciting automatic imitation [11], [27] or an interference effect [12]. However, the present findings show a common linear trend for motor responses after dot and demonstrator observation, providing evidence that imitation performance did not vary when a dot or a human moved in front of the participants. Further, the Kinematic experiment showed that observation of a non-biological stimulus did not contaminate the participants' movement velocities. Together these results strongly supported that biological kinematics induced automatic imitation. In this regards, Watanabe [22] pointed out the fundamental role played by the biological kinematics of a moving stimulus to induce a behavioural speed contagion. Thus, in our case, irrespective of whether the stimulus was artificially or human generated, the observed biological kinematics equally contaminated subsequent actions. A similar dominance of movement kinematics over the nature of the agent in action perception has been shown for apparent motion in Grosjean et al. study [28], where they proved the Fitt's Law holds for action perception of both biological and non-biological agents. Interestingly, Stanley et al. [29] found an interference effect in participant motion when both a biological and a non-biological dot was described as human generated, but not when computer generated. A potential reason for this discrepancy is that when information on the origin of the stimulus is lacking (as in the present experiment), the observer might automatically attribute a biological origin to the dot motionwhen it moves with biological kinematics, and a non-biological origin when it moves with non-biological kinematics.

### Underestimation of the observed movement velocity during explicit imitation

In explicit task, the results show that the observers' slowest and fastest pointing movements were always preceded by slow (S) and fast (F) stimuli, respectively, in agreement with implicit results. This indicates the participants' ability to distinguish stimulus velocities (perceptual ability) and their attempt to replicate them (motor ability), though they fail in exact reproduction. The statistical analysis confirmed the expected differences between the tasks. When participants were explicitly instructed to imitate the stimulus velocity, imitation performance improved probably as a result of greater attention directed toward the kinematic features of the stimulus. However, in explicit conditions, the mechanism immediately translating the observed motion into the produced one showed limitations.

Biomechanical constraints that reduce velocity during the execution of fast arm movement are one possible explanation for the participants' incapacity to reproduce the observed motion velocity exactly. However, this hypothesis seems unacceptable for several reasons. First, the demonstrator was actually able to produce high pointing-movement velocities. Second, in order to verify the participants' motor capacities to produce rapid movements they were asked to point as fast as possible. The recorded velocity values were all in the range of the velocities performed by the demonstrator or displayed on the screen.

Inaccuracy in movement duration estimation may also explain the systematic trend to produce movements slower than the stimulus ones. In the present experiment, subjects were not asked to reproduce movement duration but to imitate the displayed velocity. However, an estimation process of stimulus duration on the basis of its velocity and amplitude cannot be rejected. Several studies have demonstrated that movement duration (time interval tested 200 ms – 2 s) is systematically overestimated [30], [31]. Accordingly, the present displayed duration might have been linearly overestimated for the time interval that was tested (300 ms – 1.3 s). This possibility is, however, restricted by the fact that, for the visual stimuli tested here, time estimation should have relied on successive acceleration and deceleration phases that are more challenging in terms of cognitive demand compared to a constant velocity stimulus.

More plausibly, perceptual inaccuracy of the visual system to measure acceleration can explain the imitation degradation that was found linearly to be velocity-dependent. Additionally, the mechanisms involved in translating the perceived features of a stimulus into a motor command could also degrade a participant's imitation capacity. During an explicit imitation, the motor plan had to produce the finger's specific trajectory and velocity based on the observed trajectory and velocity. Moreover, noise could be generated during the transformation of the visual input into the motor imitation as is the case during action production [32], and the amount of noise could be proportional to the magnitude of the stimulus velocity.

Hence, even if the present study indicated the motor effect of the observed action and confirmed a strong link between perceptual and action systems, both automatically and voluntarily, a perfect matching between the perceived movement and the observer's motor performance is not possible, even for a simple arm pointing task. This is not in opposition to the direct-matching hypothesis, but it puts in perspective a *stricto sensu* utilization of the term *direct*.

### Conclusion

The linear trend observed both in implicit and in explicit conditions (Figure 3) suggests the existence of imitation processes that are largely automatic and independent of the visual context (dot or human model). Implicit imitation could represent the initial critical sensorimotor step on the basis of which higher levels of social interaction behaviours are built, through a combination of these low-level units. In support of this idea, we found that movement kinematics influenced subsequent action, and the observation-matching system was insensitive to human vs. computer generated stimuli. An interesting proposal is that a poor display, moving with biological kinematics, could aid in recognizing the actions of others and, thereby in rapidly apprehending their mental states. Yet, why is implicit imitation dependent upon kinematics? It has been proposed that motor memory and internal models of action are stored in terms of kinematic parameters and that complex actions are a composition of kinematic subunits, or motor primitives [33]. Consequently, when visual information is missing, an efficient recognition of complex behaviour would be dependent upon an individual's capacity to discriminate kinematic parameters.
